# Syndecan 4 Upregulation on Activated Langerhans Cells Counteracts Langerin Restriction to Facilitate Hepatitis C Virus Transmission

**DOI:** 10.3389/fimmu.2020.00503

**Published:** 2020-03-27

**Authors:** Bernadien M. Nijmeijer, Julia Eder, Catharina J. M. Langedijk, Tanja M. Kaptein, Sofie Meeussen, Pascale Zimmermann, Carla M. S. Ribeiro, Teunis B. H. Geijtenbeek

**Affiliations:** ^1^Department of Experimental Immunology, Amsterdam Infection and Immunity Institute, Amsterdam University Medical Centers, University of Amsterdam, Amsterdam, Netherlands; ^2^Department of Human Genetics, KU Leuven, Leuven, Belgium; ^3^Centre de Recherche en Cancérologie de Marseille, Equipe labellisée Ligue 2018, Aix-Marseille Université, Inserm, CNRS, Institut Paoli Calmettes, Marseille, France

**Keywords:** langerhans cells, hepatitis C virus, heparan sulfate proteoglycans, heparan sulfates, syndecan 4, langerin, viral dissemination

## Abstract

Sexually transmitted Hepatitis C virus (HCV) infections and high reinfections are a major concern amongst men who have sex with men (MSM) living with HIV-1 and HIV-negative MSM. Immune activation and/or HIV-1 coinfection enhance HCV susceptibility via sexual contact, suggesting that changes in immune cells or external factors are involved in increased susceptibility. Activation of anal mucosal Langerhans cells (LCs) has been implicated in increased HCV susceptibility as activated but not immature LCs efficiently retain and transmit HCV to other cells. However, the underlying molecular mechanism of transmission remains unclear. Here we identified the Heparan Sulfate Proteoglycan Syndecan 4 as the molecular switch, controlling HCV transmission by LCs. Syndecan 4 was highly upregulated upon activation of LCs and interference with Heparan Sulfate Proteoglycans or silencing of Syndecan 4 abrogated HCV transmission. These data strongly suggest that Syndecan 4 mediates HCV transmission by activated LCs. Notably, our data also identified the C-type lectin receptor langerin as a restriction factor for HCV infection and transmission. Langerin expression abrogated HCV infection in HCV permissive cells, whereas langerin expression on the Syndecan 4 expressing cell line strongly decreased HCV transmission to a target hepatoma cell line. These data suggest that the balanced interplay between langerin restriction and Syndecan 4 transmission determines HCV dissemination. Silencing of langerin enhanced HCV transmission whereas silencing Syndecan 4 on activated LCs decreased transmission. Blocking Heparan Sulfate Proteoglycans abrogated HCV transmission by LCs *ex vivo* identifying Heparan Sulfate Proteoglycans and Syndecan 4 as potential targets to prevent sexual transmission of HCV. Thus, our data strongly suggest that the interplay between receptors promotes or restricts transmission and further indicate that Syndecan 4 is the molecular switch controlling HCV susceptibility after sexual contact.

## Introduction

Viral hepatitis is responsible for an estimated 1.3 million deaths from acute infection, hepatitis-related liver cancer and cirrhosis in 2015 (1). Hepatitis C virus (HCV) infections accounts for almost 30% of these deaths (1). In the mid-2000s HCV infection emerged in men who have sex with men (MSM) (2–5) likely due to sexual contact (6–9). Although directly acting antiviral (DAA) treatment is very effective in clearing HCV (10), the high reinfection rates amongst MSM who cleared HCV spontaneously or who were successfully treated (11–13) underscores the need for a better understanding of the mechanisms involved in sexual transmission of HCV. As new HCV infections were typically found in MSM living with human immunodeficiency virus type 1 (HIV-1), it was suggested that the HIV-1 status is an important risk factor for sexually acquired HCV (8, 9, 11, 14). However, recent studies suggest that sexual transmission of HCV also occurs in HIV-1-negative MSM eligible for or using pre-exposure prophylaxis (PrEP), indicating that HIV-1 infection status is not the only factor affecting susceptibility (15–17). Potential mechanisms for increased rates of sexual transmission of HCV among MSM may include high-risk practices (4) and unprotected mucosal traumatic sex leading to rectal bleeding (18) which could lead to disruption of the mucosal integrity allowing HCV to cross the epithelial barrier to either directly enter the blood stream or indirectly via immune cells promoting sexual transmission of HCV. We have previously shown an important role for Langerhans cells (LCs) in HCV transmission during HIV-1 coinfection but also upon immune activation (19).

LCs reside in mucosal tissues and are therefore among the first immune cells to encounter viruses, they are thought to be involved in sexual transmission of HIV-1 (20–22) Interestingly, LC function in HIV-1 infection depends on their activation state (20–22). Immature LCs have been shown to restrict HIV-1 infection via the C-type lectin receptor langerin (CD207) (20, 21). Langerin is highly expressed by immature LCs and capture of HIV-1 by langerin leads to viral internalization into Birbeck granules. Virus fusion triggers degradation of HIV-1 via E3-ubiquitin ligase tri-partite-containing motif 5α (TRIM5α) mediated autophagic degradation, thereby preventing infection of LCs (20, 21). Upon LC activation, as can occur during genital coinfections, LCs lose their protective function, become infected by HIV-1 and efficiently transmit HIV-1 to T cells, thereby promoting HIV-1 dissemination (22).

Primary LCs are also present at mucosal sites of the foreskin and within the anal tissue (19, 23), the primary entry site of sexually transmitted HCV. Interestingly, under normal conditions LCs are refractory to HCV but upon HIV-1 infection or when activated LCs are able to retain HCV, facilitating transmission to target cells (19). These data suggest that activation of LCs in MSM might allow the virus to penetrate mucosal tissues and establish dissemination via the blood or lymph (24). Thus, the activation state of the LCs dictates HCV susceptibility but the molecular mechanism deciding the fate of the virus are unknown. Therefore we have investigated the molecular mechanism involved in HCV susceptibility during sexual contact. Syndecans are known to function as attachment receptors to facilitate HIV-1 transmission (25, 26). Syndecans are transmembrane heparan sulfate proteoglycans (HSPGs) expressed on the surfaces of human cells that possess heparan sulfate glycosaminoglycan chains (27). They are classified into members of the Syndecan family that consist of Syndecan 1, Syndecan 2, Syndecan 3, and Syndecan 4 (28, 29). Sexually transmitted viruses such as HIV-1, herpes simplex virus type 1 and 2 (HSV-1 and HSV-2) and human papillomavirus (HPV) interact with heparan sulfates on Syndecans to mediate binding and internalization to host cells to promote infection and spread to other cells (30–35). HCV also interacts with heparan sulfates on Syndecan 1 and Syndecan 4 which can facilitate attachment and infection in cell lines (36, 37).

Here our data show that upon activation LCs acquire the ability to retain and transmit HCV using HSPGs. Further analyses showed that the major HSPG involved is Syndecan 4. Strikingly, Syndecan 4 induction upon activation of LCs counteracts langerin restriction of HCV transmission. Ectopic expression of langerin on the Syndecan 4 expressing cell line enhanced HCV capture but decreased HCV transmission to a target hepatoma cell line Huh 7.5. Moreover, silencing langerin on Mutz-LCs enhanced HCV transmission which strongly support a restrictive role for langerin in mucosal HCV transmission. Together our data strongly suggest that activated LCs upregulate Syndecan 4 to capture HCV via their heparan sulfate chains and exploit them as in *trans*-receptors to infect hepatocytes. This transmission mechanism implicates that the balanced interplay between langerin and Syndecan 4 on activated LCs dictates HCV susceptibility after sexual contact. Therapeutical interventions targeting Syndecan 4 on mucosal LCs could represent a novel strategy to counteract LC-mediated dissemination of HCV.

## Materials and Methods

### Antibodies and Reagents

The following antibodies were used (all anti-human): Heparan Sulfate (clone F58-10E4) (Amsbio), digested Heparan (clone F69-3G10) (Amsbio), Syndecan 1 (DL-101) (Santa Cruz), Syndecan 2-FITC (H-7) (Santa Cruz), Syndecan 3 (M-300) (Santa Cruz), Syndecan 4 (clone F94-8G3), CD207-PE (langerin) mouse IgG1 (#IM3577) (BeckmanCoulter, USA), CD1a-APC mouse IgG1 (BD Biosciences, San Jose, CA, USA) CD1a-PE (clone SK9) mouse IgG2b (BD Bioscience), HLA-B27-FITC (clone HLA-ABC-m3), mouse IgG2a (Abcam), HLA-DR-FITC (clone G46-6), mouse IgG2b (BD Bioscience), CD80-PE, mouse IgG1 (BD Pharmingen), CD83-PE, mouse IgG1 (eBioscience), CD86-FITC, mouse IgG1 (BD Pharmingen), FITC-conjugated goat-anti-mouse IgM (#31992) (Invitrogen), AF488-conjugated goat-anti-mouse IgG1 (#A21121) (Invitrogen), AF488-conjugated donkey-anti-mouse IgG2b (Invitrogen).

The following reagents were used: Unfractionated (UF) heparin, 5.000 I.E./ml (LEO). Low Molecular Weight (LMW) heparins: dalteparin, 10.000 IE anti-Xa/ml (Pfizer), tinzaparin, 10.000 IE anti-X1/0.5ml (LEO), enoxaparin, 100 mg/ml (Sanofi). 4-Nitrophenyl β-D-xylopyranoside (PNP-Xyl, 2001-96-9) (SigmaAldrich). Heparinase III from *Flavobacterium heparium*, EC 4.2.2.8, Batch 010 (Amsbio). 123Count eBeads, REF# 01-1234-42, LOT# E133305, 1.011.000 eBeads/ml (eBioscience).

### Plasmids and Viruses

The following plasmids were provided by Dr. Takaji Wakita at Tokyo Metropolitan Institute of Neuroscience: Genotype 2a HCV genomic RNA clone pJFH1 (APP1025) (38). pNL4.3.Luc_RΔenv provided by Dr. N.R. Landau (39), pHCV_H77_E1_E2(AF009606) Dr. Joe Grove (Addgene) (40). For single-round infection assay, human embryonic kidney 293T/17 cells (ATCC, CRL-11268) were co-transfected with pNL4.3.Luc_RΔenv, containing firefly luciferase gene at the *nef* position (1.35 ug) and genotype 1a pHCV_H77_E1_E2(AF009606) (0.6 ug). Transfection was performed in 293T/17 cells using genejuice (Novagen, USA) transfection kit according to manufacturer's protocol. At day 3 or day 4, pseudotyped HCV virus particles were harvested and filtered over 0.45 um nitrocellulose membrane (SartoriusStedim, Gottingen, Germany). Replicative JFH1-AM120-Rluc *in vitro* transcribed RNA, containing a luciferase reporter gene, was generated according to manufacturer's instructions (Ambion MEGAscript-kit, ThermoFisher, USA) and electroporated into Huh 7.5 cells as previously described (41). Virus particles were harvested on day 8 and, TCID50s were determined. The TCID50 of HCV ranged from 2 × 10^3^ to 4 × 10^3^.

### Cell Lines

The human B cell line Namalwa (ATCC, CRL-1432) and Namalwa cells stably expressing human Syndecan 1, Syndecan 2, Syndecan 3, Syndecan 4 [described earlier, laboratory Prof. Zimmermann (42)] were maintained in RPMI 1640 medium (Gibco Life Technologies, Gaithersburg, Md.) containing 10% fetal calf serum (FCS)The expression of the different Syndecans was validated by flow cytometry using core protein-specific antibodies directed against the different Syndecans. Huh 7.5 (human hepatocellular carcinoma) cell line were provided by dr. Charles M. Rice (43). Cells were maintained in Dulbecco modified Eagle medium (Gibco Life Technologies, Gaithersburg, Md.) containing 10% fetal calf serum (FCS) and penicillin/streptomycin. Medium was supplemented with 1mM Hepes buffer (Gibco Life Technologies, Gaithersburg, Md.). Mutz-LCs were differentiated from CD34^+^ human AML cell line Mutz3 progenitors in the presence of GM-CSF (100 ng/ml, Invitrogen), TGF-β (10 ng/ml, R&Dsystems) and TNF-α (2.5 ng/ml), R&Dsystems) and cultured as described before (44). Cell surface expression of heparan sulfates and Syndecan 4 on Mutz-LCs was verified by flow cytometry using antibodies directed against CD1a, CD207 and respectively heparan sulfate or Syndecan 4. Flow cytometric analyses were performed on a BD FACS Canto II (BD Biosciences). Data was analyzed using FlowJo vX.0.7 software (TreeStar).

### Huh 7.5 and Namalwa Cell Line Langerin Transduction

Langerin expression plasmid pcDNA3.1 were obtained from Life Technologies and subcloned into lentiviral construct pWPXLd (Addgene). HIV-1-based lentiviruses were produced by co-transfection of 293T cells with the lentiviral vector construct, the packaging construct (pPAX2, Addgene) and vesicular stomatitis virus glycoprotein envelope (pMD2.G, Addgene) as described previously (45). Huh 7.5 cell line or Namalwa Syndecan 4 cell line were transduced with HIV-1-based lentiviruses expressing sequences coding human wild-type langerin. Subsequently cells were sorted using a FACS Aria (BD) based on CD207-PE mouse IgG1 (#IM3577). Ectopic expression of langerin was confirmed by flow cytometry.

### *Ex vivo* Model and Primary LC Isolation

Epidermal sheets were prepared as described previously (20, 46). Briefly, skin-grafts were obtained using a dermatome (Zimmer Biomet, Indiana USA). After incubation with Dispase II (1 U/ml, Roche Diagnostics), epidermal sheets were separated from dermis, washed, cut in 1 cm^2^ and cultured in Iscoves Modified Dulbeccos's Medium (IMDM, Thermo Fischer Scientific, USA) supplemented with 10% FCS, gentamicin (20 μg/ml, Centrafarm, Netherlands), penicillin/streptomycin (10 U/ml and 10 μg/ml, respectively; Invitrogen). Activated LCs were generated as described before (20). Briefly, obtained epidermal sheets were separated from dermis, washed and cultured in IMDM (Thermo Fischer Scientific, USA) supplemented with 10% FCS, gentamicin (20 μg/ml, Centrafarm, Netherlands), penicillin/streptomycin (10 U/ml and 10 μg/ml, respectively; Invitrogen) for 3 days and activated LCs were harvested. Immature LC-enriched epidermal single-cell suspensions were generated as described before (20, 46). Briefly, epidermal sheets were incubating in PBS containing DNase I (20 units/ml; Roche Applied Science) and trypsin 0.05% (Beckton Dickinson, USA). Single-cell suspension was layered on Ficoll gradient (Axis-shield) and immature LCs were purified using CD1a microbeads (Miltenyi Biotec, Germany). LCs were routinely 85 to 98% pure and expressed high levels of Langerin and CD1a (22). Cell surface expression of heparan sulfates on primary LCs was verified by flow cytometry using antibodies directed against CD207 (langerin) and CD1a and heparan sulfates for immature LCs and CD1a and heparan sulfates for activated LCs.

### Transmission Assays and Co-culture

Namalwa cell line (1.0 × 10^6^ cells/ml, 100 ul per well) or Namalwa Syndecan 1-4 cell line (1.0 × 10^6^ cells/ml, 100 ul per well) or immature LCs (8.0 × 10^5^ cells/ml and 1.0 × 10^6^ cells/ml LCs, 100 ul per well) or activated LCs (1.0 × 10^6^ cells/ml LCs, 100 ul per well) were exposed to replicative HCV genotype 2a strain containing a luciferase reporter gene (JFH1-AM120-Rluc) or pseudotyped HCV (HIV-1 NL4.3Δenv pseudotyped with HCV env glycoproteins E1 and E2) pre-incubated with 250 U or 500 U UF heparin or LMW heparins either for 4 or 24 h, harvested, extensively washed to remove unbound virus and co-cultured with Huh 7.5 for 3 or 5 days at 37°C and analyzed for luciferase reporter activity. Luciferase activity (relative light units (R.L.U.)) was measured using the Luciferase assay system (Promega, USA) or Reporter gene assay system (Britelite plus, PerkinElmer) according to manufacturer's instructions. For the *ex vivo* transmission model epidermal sheets were culture for 24 h and exposed to medium or UF heparin pre-incubated replicative HCV or pseudotyped HCV for another 24 h. After 48 h cells were harvested, extensively washed to remove unbound virus and co-cultured with Huh 7.5 for 5 days at 37°C, transmission was determined by luciferase reporter activity. Luciferase activity (relative light units (R.L.U.)) was measured using the Luciferase assay system (Promega, USA) or Reporter gene assay system (Britelite plus, PerkinElmer) according to manufacturer's instructions.

### RNA Interference

MUTZ-LCs and primary LCs were silenced by electroporation with Neon Transfection System (ThermoFischer Scientific). The siRNA were specific for langerin (10 μM siRNA, M-013059-01, SMARTpool; Dharmacon), siRNA Syndecan 4 (10 μM siRNA, M-003706-01-0005, SMARTpool; Dharmacon) or non-targeting siRNA (D-001206-13, SMARTpool; Dharmacon) as control. Silencing of the targets was verified by real-time PCR, flow cytometry. Cells were used for experiment 72 h after silencing. Silencing of the targets was verified by real-time PCR or flow cytometry.

### RNA Isolation and Quantitative Real-Time PCR

mRNA was isolated with an mRNA Capture kit (Roche) and cDNA was synthesized with a reverse-transcriptase kit (Promega) and PCR amplification was performed in the presence of SYBR green in a 7500 Fast Realtime PCR System (ABI). Specific primers were designed with Primer Express 2.0 (Applied Biosystems). Primer sequences used for mRNA expression were for gene product: GAPDH, forward primer (CCATGTTCGTCATGGGTGTG), revers primer (GGTGCTAA GCAGTTGGTGGTG). For gene product: langerin, forward primer (CACAGTGGCATTCTGGAGTCC), reverse primer (CCACCCCTCCCACTTTAACC). For gene product: Syndecan 4, forward primer (AGGTGTCAATGTCCAGCACTGTG) reverse primer (AGCAGTAGGATCAGGAAGACGGC). The normalized amount of target mRNA was calculated from the Ct values obtained for both target and household mRNA with the equation Nt = 2^∧^Ct(GAPDH) – Ct(target). For relative mRNA expression, control siRNA sample was set at 1 for each donor.

### Biosynthesis Inhibition and Enzymatic Treatment

Namalwa Syndecan 4 cell line were cultured in the presence of 1.0 mM or 2.5 mM PNP-Xyl for 72 h to inhibit HSPG biosynthesis and used in subsequent experiments. The expression of cell surface heparan sulfates was assessed by flow cytometry using antibodies directed against heparan sulfates. 1.0 × 10^6^ cells/ml were treated in D-PBS/0.25% BSA with 140 miliunits heparinase III (Amsbio) for 2 h at 25°C, washed and used in subsequent experiments. Enzymatic digestion was verified by flow cytometry using antibodies directed against heparan sulfates and digested heparan sulfates.

### Statistics

A two-tailed, parametric Student's *t*-test for paired observations (differences within the same donor) or unpaired observation (differences between different donors) was performed. For unpaired, non-parametric observations a Mann-Whitney test was performed. Statistical analyses were performed using GraphPad Prism 7 software and significance was set at ^*^P < 0.05, ^**^P < 0.01, ^***^P < 0.001, ^****^P < 0.0001.

## Results

### Heparan Sulfate Proteoglycans Promote HCV Transmission

**Figure 1:**
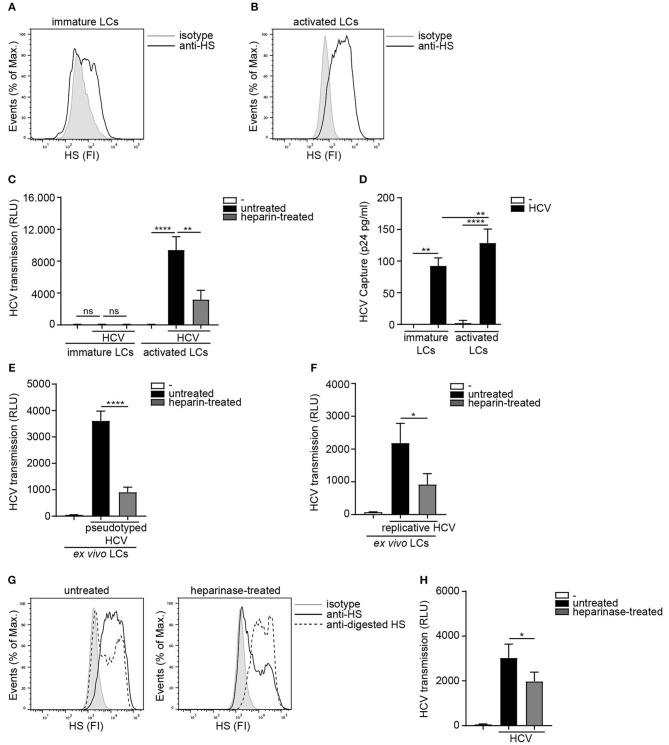
Heparan sulfate proteoglycans promote HCV transmission. **(A,B)** Immature and activated LCs express heparan sulfates. One representative donor out of 3 is depicted. **(C)** Pseudotyped HCV was pre-incubated with heparin (250U) and transmission by LCs to Huh 7.5 cells was determined. **(D)** Immature and activated LCs were exposed to pseudotyped HCV, lysed and binding was quantified by p24 ELISA. **(E,F)** *Ex vivo* tissue was cultured in medium for 24 h and subsequently exposed to either medium or **(E)** heparin (500 U) pre-incubated pseudotyped HCV or pseudotyped HCV for another 24 h and transmission by LCs to Huh 7.5 cells was determined. **(F)** heparin (500 U) pre-incubated replicative HCV or replicative HCV for another 24 h and transmission by LCs to Huh 7.5 cells was determined. **(G)** Activated LCs were left untreated or treated with heparinase for 2 h and heparan sulfate expression was determined. One representative donor out of 3 is depicted. **(H)** Activated LCs were left untreated or treated with heparinase for 2 h, washed, exposed to pseudotyped HCV and transmission by LCs to Huh 7.5 cells was determined. Error bars are the mean ± SD of **(C)** immature LCs *n* = 3 donors measured in triplicate or activated LCs *n* = 3 donors measured in triplicate. **(D)** immature LCs *n* = 3 donors measured in duplicate or activated LCs *n* = 7 donors measured in duplicate. **(E)** *n* = 3 donors measured in quadruplicate. **(F)** *n* = 4 donors measured in quadruplicate. **(H)** *n* = 3 donors measured in quadruplicate. ns = not significant, *p < 0.05, **p < 0.01, ****p < 0.0001 by two-tailed, unpaired, non-parametric, Mann-Whitney test. LCs: Langerhans cells, HS: heparan sulfates, FI: fluorescent intensity, RLU: relative light units, HCV: Hepatitis C virus.

As HSPGs are known receptors for HCV on hepatocytes (36, 37) we investigated the role of heparan sulfates in LC-mediated HCV transmission. Immature and activated LCs were isolated and their phenotype was verified (20) (Supplemental Figure S1). Activated in contrast to immature LCs expressed high levels of heparan sulfates on their cell surface (Figures 1A,B). Next we blocked the heparan sulfate binding places on HCV using heparin to investigate their role in transmission. Notably, heparin treatment of pseudotyped HCV (HIV-1 NL4.3Δenv pseudotyped with HCV env glycoproteins E1 and E2) blocked HCV transmission by activated LCs (Figure 1C). Immature LCs did not transmit HCV to target cells (Figure 1C), even though both, immature and activated LCs captured HCV (Figure 1D). Moreover, heparin treatment of both pseudotyped HCV genotype 1a strain pHCV_H77_E1_E2 and replicative HCV genotype 2a strain (JFH1-AM120-Rluc) blocked HCV transmission in the *ex vivo* transmission model (Figures 1E,F) confirming a role for heparan sulfates on *ex vivo* LCs in HCV transmission. Next we removed heparan sulfates from activated LCs by enzymatic digestion to investigate the involvement of HSPGs. Enzymatic treatment of activated LCs with heparinase decreased the expression of heparan sulfates on the cell surface (Figure 1G). Notably, heparan sulfate removal resulted in a significant decrease of HCV transmission, compared to untreated LCs (Figure 1G). These data strongly suggest that HSPGs expressed by LCs, mediate HCV transmission.

### Syndecan 4 Facilitates HCV Transmission

**Figure 2:**
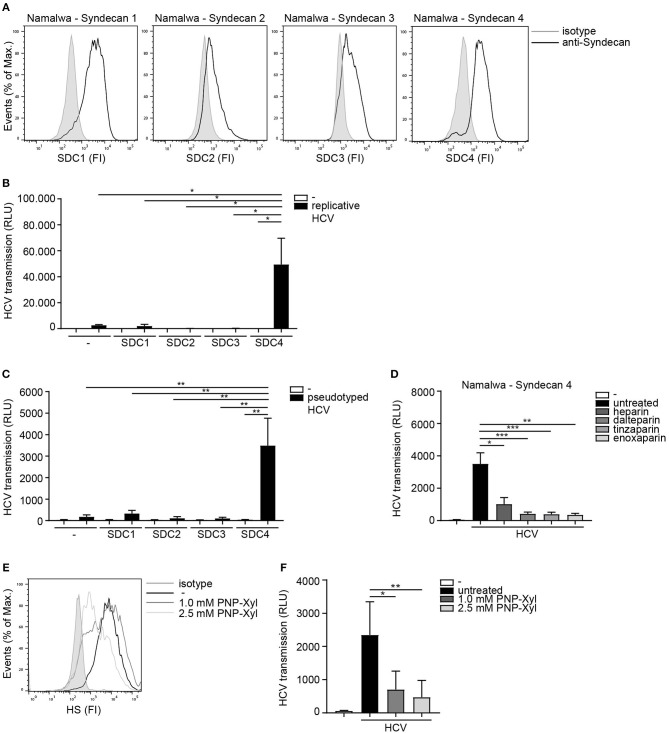
Syndecan 4 facilitates HCV transmission. **(A)** Different Syndecan cell lines express Syndecan 1-4, on the cell surface determined by flow cytometry. One representative experiment out of 2 is depicted. **(B,C)** Different Syndecan cell lines were exposed to **(B)** replicative HCV or **(C)** pseudotyped HCV **(B,C)** and transmission by LCs to Huh 7.5 cells was determined. **(D)** Pseudotyped HCV was pre-incubated with unfractionated heparin (500U) or dalteparin (500U) or tinzaparin (500U) or enoxaparin (500U) and transmission by LCs to Huh 7.5 cells was determined. **(E)** Syndecan 4 cells were cultured in the presence of PNP-Xyl inhibitor for 72 h and heparan sulfate surface expression was determined by flow cytometry. One representative donor out of 3 is depicted. **(F)** Syndecan 4 cells were cultured in the presence of PNP-Xyl inhibitor for 72 h, harvested, exposed to pseudotyped HCV and transmission by Syndecan 4 cells to Huh 7.5 cells was determined. Error bars are the mean ± SD of **(B)** one representative donor measured in quadruplicate. **(C)** one representative donor out of 3 measured in quadruplicate. **(D)** *n* = 5 experiments (medium and HCV) or *n* = 3 experiments (heparin, dalteparin, tinzaparin, enoxaparin) measured in triplicate or quadruplicate. **(F)** *n* = 5 experiments measured in quadruplicate. *p < 0.05, **p < 0.01, ***p < 0.001, by two-tailed, unpaired, parametric, Student *t*-test. SDC1: Syndecan 1, SDC2: Syndecan 2, SDC3: Syndecan 3, SDC4: Syndecan 4, PNP-Xyl: p-Nitrophenyl-β-d-xylopyranoside, HS: heparan sulfates, FI: fluorescent intensity, RLU: relative light units, HCV: Hepatitis C virus.

Next we assessed the potential role for HSPGs to serve as HCV transmission receptors. We investigated the ability of human B cell line Namalwa expressing the different Syndecans (Figure 2A) to transmit HCV. Only the cell-line expressing Syndecan 4 was able to transmit replicative HCV genotype 2a strain to target cells (Figure 2B). Similarly, Syndecan 4 also efficiently transmitted pseudotyped HCV in contrast to the other Syndecans (Figure 2C). These data strongly suggest that HCV specifically interacts with Syndecan 4 for transmission. Next we examined the role of heparan sulfates in HCV transmission by Syndecan 4 cells using not only unfractionated heparin, but also several low molecular weight (LMW) heparins. Pseudotyped HCV was exposed to either unfractionated heparin or LMW heparins dalteparin, tinzaparin and enoxaparin and HCV transmission was determined. The different LMW heparins inhibited transmission by Syndecan 4 to a similar extent as unfractionated heparin (Figure 2D), strongly suggesting that heparan sulfates on Syndecan 4 are important for the interaction with HCV. Finally we inhibited heparan sulfate biosynthesis by PNP-Xyl treatment. Syndecan 4 cells were left untreated or cultured in the presence of 1.0mM or 2.5mM PNP-Xyl for 72 h. The cell viability was not affected at these concentrations (data not shown). PNP-Xyl treatment decreased heparan sulfate expression on the cell surface of Syndecan 4 cells (Figure 2E), and abrogated HCV transmission in a concentration dependent manner (Figure 2F). Thus, our data strongly suggest that Syndecan 4 in contrast to the other Syndecans is a specific receptor for HCV transmission.

### Activated LCs Transmit HCV via Syndecan 4

**Figure 3:**
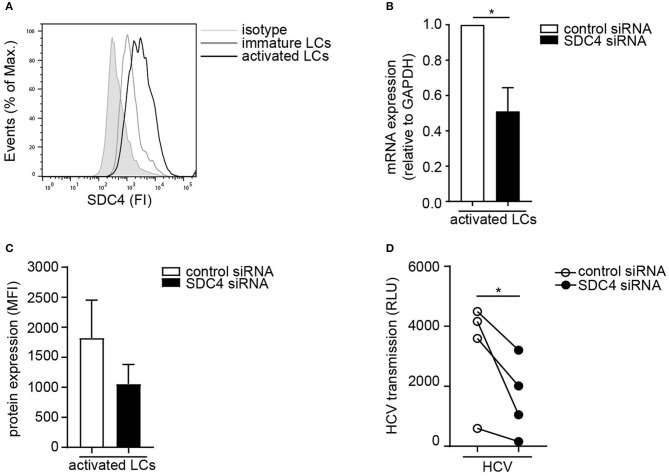
Activated LCs transmit HCV via Syndecan 4. **(A)** Immature and activated LCs express Syndecan 4 on the cell surface. One representative donor out of 2 is depicted. **(B)** Syndecan 4 silencing was confirmed by real-time PCR. mRNA expression was normalized to GAPDH and set at 1 in cells treated with control siRNA. **(C)** Cell surface expression of Syndecan 4 after silencing determined by flow cytometry**. (D)** Activated LCs were transfected with non-target control or Syndecan 4 siRNA and after 72 h exposed to pseudotyped HCV and transmission by LCs to Huh 7.5 cells was determined. Error bars are the mean ± SD of **(B)** *n* = 4 donors, **(C)** *n* = 3 donors, **(D)** *n* = 4 donors measured in triplicate or quadruplicate. *p < 0.05, by two-tailed, paired, parametric, Student *t*-test. LCs: Langerhans cells, SDC4: Syndecan 4, HS: heparan sulfates, FI: fluorescent intensity, MFI: mean fluorescent intensity, RLU: relative light units, HCV: Hepatitis C virus.

Next we investigated the Syndecan 4 expression on primary LCs. Activated LCs expressed higher levels of Syndecan 4 than immature LCs from the same donor (Figure 3A). In order to investigate the role of Syndecan 4 in transmission by LCs, Syndecan 4 was silenced in primary activated LCs by RNA interference (Figures 3B,C). Silencing Syndecan 4 did not interfere with the expression of other Syndecans or langerin (Supplemental Figure S2). Syndecan 4 silencing strongly decreased HCV transmission by activated LCs compared to the non-targeting control (Figure 3D). Thus, our data indicate that Syndecan 4 expression is upregulated upon activation of LCs and thereby facilitates HCV transmission to hepatocytes.

### Langerin Restricts HCV Infection

**Figure 4:**
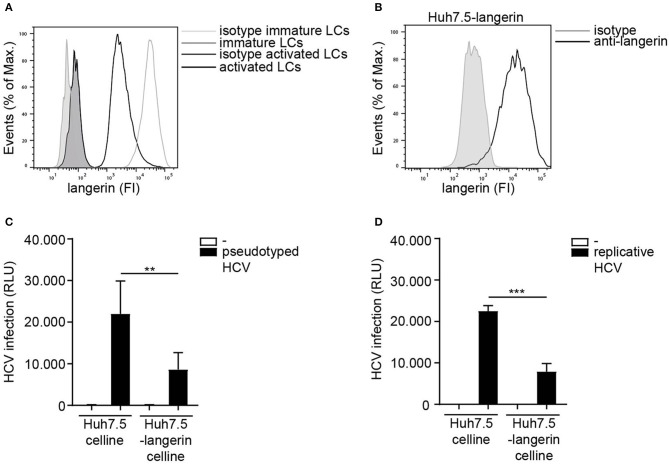
Langerin restricts HCV infection. **(A)** Immature and activated LCs from the same donor express langerin. One representative donor out of 3 is depicted. **(B)** Huh 7.5-langerin cell line was transduced with lentiviruses expressing sequences coding human langerin. Surface expression was determined by flow cytometry. One representative experiment is depicted. **(C,D)** Huh 7.5 cell line or Huh 7.5-langerin cell line was infected with **(C)** pseudotyped HCV or **(D)** replicative HCV **(C,D)** and cell line infection was determined. Error bars are the mean ± SD of **(C)** *n* = 6 experiments measured in triplicate or quadruplicate. **(D)** *n* = 3 experiments measured in triplicate. **p < 0.01, ***p < 0.001, by two-tailed, unpaired, parametric, Student *t*-test. FI: fluorescent intensity, RLU: relative light units, HCV: Hepatitis C virus.

LCs specifically express the CLR langerin that has a protective role in HIV-1 dissemination by restricting HIV-1 infection and transmission (20, 21). Immature LCs expressed high levels of langerin, which is downregulated upon activation of LCs (Figure 4A) (46). Nothing is known about the role of langerin in HCV infection. The Huh 7.5 cell line does not express langerin on the cell surface (Supplemental Figure S3). Therefore, we ectopically expressed langerin on the HCV susceptible Huh 7.5 cell line (Figure 4B) and investigated its function in infection. Notably, infection of langerin-expressing Huh 7.5 cells by pseudotyped HCV was significantly lower compared to Huh 7.5 cells (Figure 4C). Moreover, langerin expression also decreased infection of Huh 7.5 with replicative HCV (Figure 4D). Huh 7.5 cells and Huh 7.5-langerin cells exhibit similar growth (Supplemental Figure S4), suggesting the observed differences resulted from a decrease in infection. These data strongly suggest that langerin restricts pseudotyped as well as replicative HCV infection.

### The Interplay Between Langerin and Syndecan 4 Determines HCV Transmission

**Figure 5:**
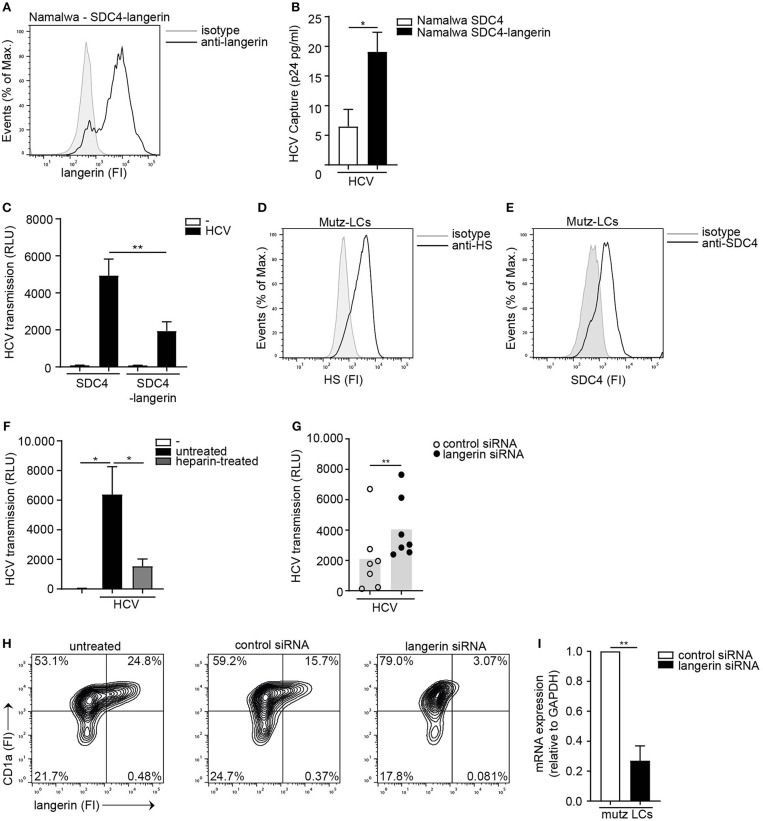
The interplay between langerin and Syndecan 4 determines HCV transmission**. (A)** Syndecan 4-langerin cell line express langerin on the cell surface determined by flow cytometry. One representative experiment is depicted. **(B)** Syndecan 4 cell line or Syndecan 4-langerin cell line, were exposed to pseudotyped HCV, lysed and binding was quantified by p24 ELISA. **(C)** Syndecan 4 cell line or Syndecan 4-langerin cell line, were exposed to pseudotyped HCV and transmission by LCs to Huh 7.5 cells was determined. **(D,E)** Mutz-LCs express **(D)** heparan sulfates **(E)** Syndecan 4 **(D,E)** on the cell surface as determined by flow cytometry. One representative donor is depicted. **(F)** Pseudotyped HCV was pre-incubated with heparin (500U) and transmission by Mutz LCs to Huh 7.5 cells was determined. **(G)** Mutz-LCs were transfected with non-target control or langerin siRNA or left untreated and after 72 h exposed to pseudotyped HCV and transmission by LCs to Huh 7.5 cells was determined. **(H)** Mutz-LCs were transfected with non-target control or langerin siRNA or left untreated and after 72 h silencing was confirmed by flow cytometry. One representative donor is depicted. **(I)** Mutz-LCs were transfected with non-target control or langerin siRNA or and after 72 h silencing was confirmed by real-time PCR. mRNA expression was normalized to GAPDH and set at 1 in cells treated with control siRNA. Error bars are the mean ± SD of **(B)** *n* = 3 experiments measured in duplicate. **(C)** *n* = 4 experiments measured in quadruplicate. **(B,C,I)** *p < 0.05, **p < 0.01, by two-tailed, unpaired, parametric, Student *t*-test. Error bars are the mean ± SD of **(F)** one representative donor out of 2, measured in quadruplicate. **(G)** *n* = 7 donors measured in triplicate. **(I)** *n* = 2 donors **(F,G)** *p < 0.05, **p < 0.01, by two-tailed, unpaired, non-parametric, Mann-Whitney test. LCs: Langerhans cells, SDC4: Syndecan 4, HS: heparan sulfates, FI: fluorescent intensity, RLU: relative light units, HCV: Hepatitis C virus.

To investigate the interplay between langerin and Syndecan 4, the Syndecan 4 Namalwa cell line was transduced with langerin (Figure 5A). Ectopic expression of langerin on the SDC4 cell line did not affect the overall expression of HS or the cell surface SDC4 expression of the cell lines (Supplemental Figure S5). Ectopic expression of langerin increased HCV capture by langerin-expressing Syndecan 4 cells (Figure 5B). Strikingly, langerin expression significantly inhibited Syndecan 4-mediated HCV transmission (Figure 5C). These data strongly suggest that langerin and Syndecan 4 have opposite roles in HCV transmission; Syndecan 4 promotes HCV transmission whereas langerin counteracts Syndecan 4 driven HCV transmission. Next we investigated the interplay between both receptors on LCs. As silencing of langerin on primary LCs is challenging, we have used Mutz-derived LCs as a validated model to study LC-mediated virus transmission (21, 44). Mutz-LCs have a more activated phenotype than immature LCs, therefore express intermediate levels of langerin (44). Concomitantly, we could confirm that Mutz-LCs also express both HSPGs and Syndecan 4 (Figures 5D,E). Mutz-LCs efficiently transmitted HCV to hepatocytes in a heparan sulfate-dependent manner, since heparin inhibited HCV transmission (Figure 5F). Langerin silencing in Mutz-LCs significantly increased HCV transmission (Figures 5G–I). Our data strongly suggest that langerin counteracts Syndecan 4-mediated transmission by LCs and therefore expression of langerin and Syndecan 4 control HCV restriction or transmission, respectively.

## Discussion

Sexually acquired Hepatitis C virus predominantly occurs amongst MSM living with HIV-1 (2–9) but also increasing cases of sexually acquired HCV have been reported amongst HIV-1 negative MSM eligible for or on PrEP (15–17, 47–49). Therefore, a better understanding of the mechanisms involved in sexual transmission of HCV are needed. Immature LCs reside in the anal mucosa and are among the first cells to encounter HCV upon receptive anal intercourse (19). We have previously shown that immature LCs are not able to transmit HCV but that either HIV-1 infection or immune activation changes the function of these LCs as activated as well as HIV-1 infected LCs retain HCV for transmission to target cells (19). Here we identified an important novel role for Syndecan 4 on activated LCs in HCV transmission. Syndecan 4 is upregulated on activated LCs facilitating HCV transmission. Moreover, we have identified a HCV restrictive role for the C-type lectin receptor langerin that is highly expressed by immature LCs and downregulated by activated LCs. Our data indicate that the interplay between langerin and Syndecan 4 on LCs controls HCV transmission and the increased expression of Syndecan 4 and simultaneous downregulation of langerin after activation of LCs might be the molecular switch allowing HCV transmission during sexual contact.

HSPGs are well known for their function as internalizing receptors or co-receptors (50). Many other viruses including, HIV-1 (51), herpesvirus (52), human papillomavirus (53), human cytomegalovirus (54), adenovirus (55), dengue virus (56), and vaccinia virus (57) use HSPG as initial binding target for transfer to a secondary receptor allowing fusion. Heparin treatment of both HCV genotype 1a as well as HCV genotype 2a blocked HCV transmission by *ex vivo* LCs, suggesting that HSPGs expressed by LCs mediate HCV transmission and that the effect is observed in multiple HCV genotypes. Moreover, Syndecan 3 has been shown to be involved in HIV-1 transmission by macrophages and DCs by supporting HIV-1 attachment, retaining viral infectivity and subsequent transmission to target cells (25, 26, 58). Initial attachment of HCV to target cells occurs via interaction with virion associated component apolipoprotein E (apoE) (59) or HCV envelope proteins E1 and E2 and HSPGs (60, 61). Especially, Syndecan 1 and Syndecan 4 have shown to be important attachment receptors for HCV on the surface of hepatocytes thereby facilitating infection (36, 37). We observed that of the different members of the Syndecan family, Syndecan 4 exclusively transmitted HCV to target cells, which was abrogated when HCV was exposed to unfractionated heparin or LMW heparins. Also, culturing Syndecan 4 cells in the presence of a heparan sulfate biosynthesis inhibitor PNP-Xyl (62–65) dose-dependently decreased HCV transmission to target cells. These data strongly suggest that the heparan sulfate or proteoglycan backbone of Syndecan 4 is different from the other Syndecans and functions as an important receptor for HCV transmission. Syndecan 4-mediated transmission of HCV was independent of infection as the B cell-line expressing Syndecan 4 was not infected with HCV (data not shown). Previously, we have shown that HCV transmission by activated LCs is also independent of infection (19). Silencing of Syndecan 4 on activated LCs decreased HCV transmission, our data confirms the importance of Syndecan 4 as attachment receptor for HCV on activated LCs, mediating HCV dissemination. In this study we have used the HCV replicon system in Huh 7.5 cells to generate replication competent virus particles. In the different models we have used the Huh 7.5 cells as target cells for transmission. Several studies have shown that hepatocytes can be infected by replicative HCV suggesting that these cells can also be used. However, primary hepatocytes retain RIG-I which would limit replication and make detection more difficult due to innate antiviral responses. Even though this study is conducted with hepatoma cell lines, Syndecan 4 is also expressed by other cells including primary hepatocytes and thereby its specific ability to transmit HCV might also be involved in virus spread in the liver via hepatocytes *in vivo*.

Activation of LCs strongly increased expression of heparan sulfates and Syndecan 4. These data suggest that Syndecan 4 has an important role in LC function not only for virus capture but also for their mobilization from mucosal sites and subsequent migration to lymphoid tissues. LCs have the ability to migrate from mucosal sites to lymphoid organs to present captured virions to permissive cells (66–68). Syndecans have been shown to play a role in cell migration and adhesion by mediating interaction of the heparan sulfate chains with various extracellular matrix proteins (69, 70). Interestingly, *ex vivo* LCs upregulate Syndecan 4 during maturation, which functionally regulates cell motility (69, 71) and our data strongly suggest that *ex vivo* LCs required Syndecan 4 for HCV transmission. Thus, upregulation of Syndecan 4 on LCs might have an important role in cellular function such as migration but is hijacked by HCV to allow dissemination during sexual contact. The retention of HCV by LCs might allow entry of virus into the blood or into lymphoid tissues where LCs might transfer the virus to other cells leading to dissemination to the liver (24). Syndecan 4 induction confers activated LCs with the ability to transmit HCV but our data suggest that this is not the only mechanism.

Immature LCs express high levels of the CLR langerin, which is downregulated upon activation. Langerin is well known for its restrictive function in HIV-1 dissemination. HIV-1 capture via langerin, leads to TRIM5α-mediated autophagic degradation of HIV-1, thereby restricting virus transmission (20, 21). Human TRIM5α is a host restriction factor that restricts retrovirus infection after viral fusion (72) and specifically in LCs, fusion of HIV-1 triggers TRIM5α-dependent autophagy restricting HIV-1 infection (21). Little is known about other viruses that are restricted by langerin. Notably, here we have identified langerin as a restriction factor for HCV infection and transmission. Ectopic expression of langerin on a HCV susceptible cell line decreased infection of replicative HCV as well as pseudotyped HCV. Furthermore, langerin prevented HCV transmission by activated LCs, and this transmission was independent of HCV infection. Hence, our study strongly support a role for mucosal LCs in restricting mucosal HCV transmission through a novel langerin-mediated mechanism. Recently, it was shown that rhesus TRIM5α restricts specific viruses from the *Flaviviridae* family via proteosomal degradation (73), suggesting that alternative TRIM5α antiviral functions and degradation pathways could be involved in langerin-mediated restriction of HCV. Further studies are required to confirm the uptake route for HCV in LCs. Interestingly, langerin also prevented HCV transmission by Syndecan 4-positive cell-lines as well as Mutz-LCs and this transmission is independent of infection. Thus, these data suggest that langerin also restricts transmission of HCV independent of fusion as we have shown previously that HCV does not infect LCs. It is possible that langerin routes HCV into langerin-induced Birbeck granules that prevent transmission or that the virus is degraded via either proteosomal degradation or autophagy.

Our data show that the interplay between Syndecan 4 and langerin on LCs controls the ability to transmit HCV by LCs. Syndecan 4 mediates transmission that is counteracted by langerin. Therefore the expression levels of both proteins are important. Immature LCs expressed low levels of Syndecan 4 whereas these cells express high levels of langerin and this might be important in preventing HCV transmission. In contrast, activation of LCs induced Syndecan 4 expression and simultaneously decreased langerin, suggesting that the change in expression patterns underlies the ability of activated LCs to transmit HCV. These data suggest that blocking Syndecan 4 function *in vivo* might limit HCV transmission in high risk populations.

Although the availability of highly effective directly acting antivirals (DAA) (10) created optimism toward HCV elimination, the incidence of acute HCV has not declined consistently due to high reinfection rates amongst MSM (11–13). Disruption of the mucosal integrity allowing HCV to cross the epithelial barrier to either directly enter the blood stream or indirectly via immune cells may promote sexual transmission of HCV since a sufficient quantity of HCV in shed into the rectum upon sexual contact (74). Our data strongly suggest an important role for both langerin and Syndecan 4 in HCV transmission by LCs. Activation of LCs leads to upregulation of Syndecan 4 which counteracts langerin restriction to facilitate viral dissemination after sexual contact. Heparin and LMW heparins could be interesting candidates to protect against sexual transmission of HCV. This concept has been investigated already in the context of other STIs such as HSV-1 and HSV-2, and HPV (30, 75–77). Interestingly, in the *ex vivo* tissue explant model where LCs reside in their natural microenvironment, blocking heparan sulfate interaction with HCV using heparin resulted in an abrogation of HCV transmission. Further investigation into the role of attachment receptors in HCV transmission would contribute to the understanding of sexual transmission of HCV in MSM.

## Data Availability Statement

The datasets generated for this study are available on request to the corresponding author.

## Ethics Statement

Human skin tissue was obtained from healthy donors undergoing corrective abdominal surgery in accordance with our institutional guidelines. This study was reviewed and approved by the Medical Ethics Review Committee of the Amsterdam University Medical Centers, location Academic Medical Center (AMC), Amsterdam, the Netherlands, reference number: W15_089 # 15.0103. All samples were handled anonymously. Written informed consent for participation was not required for this study in accordance with the national legislation and the institutional requirements.

## Author Contributions

BN performed and designed the research study, analyzed the data, and wrote the paper. JE performed the research study and analyzed the data. CL performed the research study and analyzed the data. TK performed the research study. SM contributed essential material. PZ contributed essential material. CR designed the research study and contributed essential material. TG was involved in all aspects of the study. All authors contributed to the manuscript, read, and approved the submitted version.

### Conflict of Interest

The authors declare that the research was conducted in the absence of any commercial or financial relationships that could be construed as a potential conflict of interest.
