# Supplementary figures and images for: Syndecan 4 Upregulation on Activated Langerhans Cells Counteracts Langerin Restriction to Facilitate Hepatitis C Virus Transmission
Source: Front Immunol. 2020 Mar 27;11:503. doi: 10.3389/fimmu.2020.00503 (PMC7118926; doi:10.3389/fimmu.2020.00503)

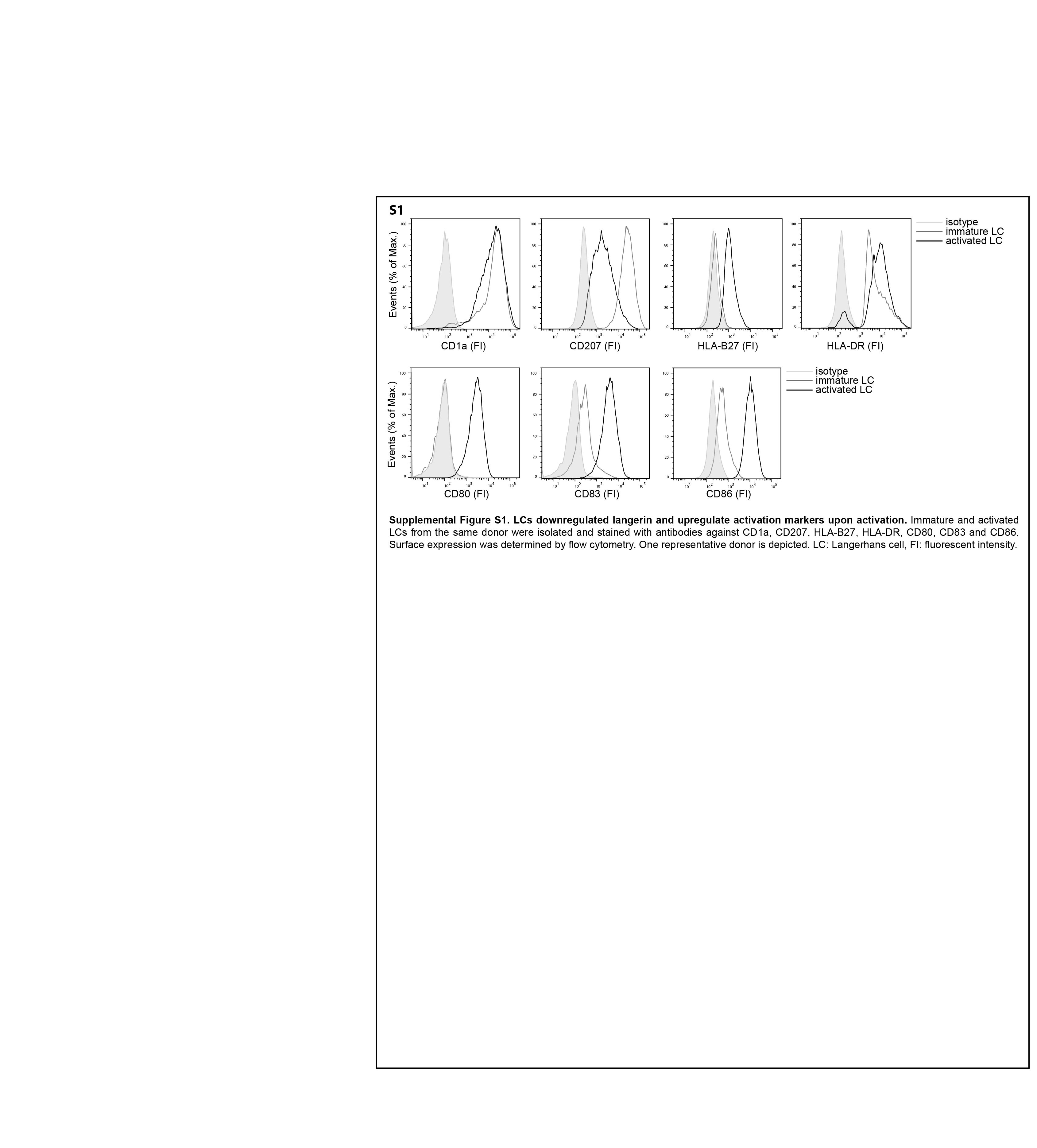

Supplement: Supplementary file 1 [file Image_1.jpg]

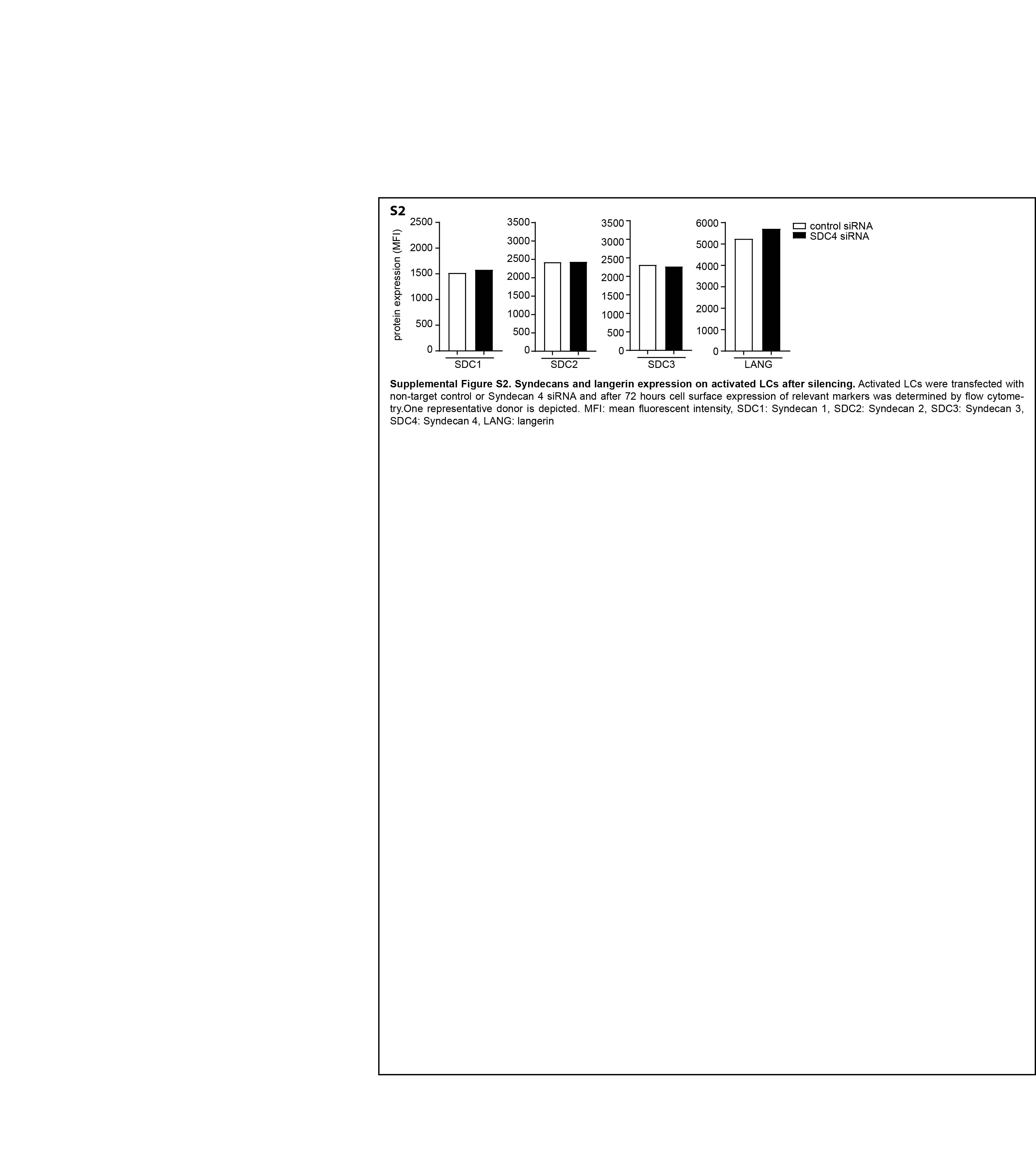

Supplement: Supplementary file 2 [file Image_2.jpg]

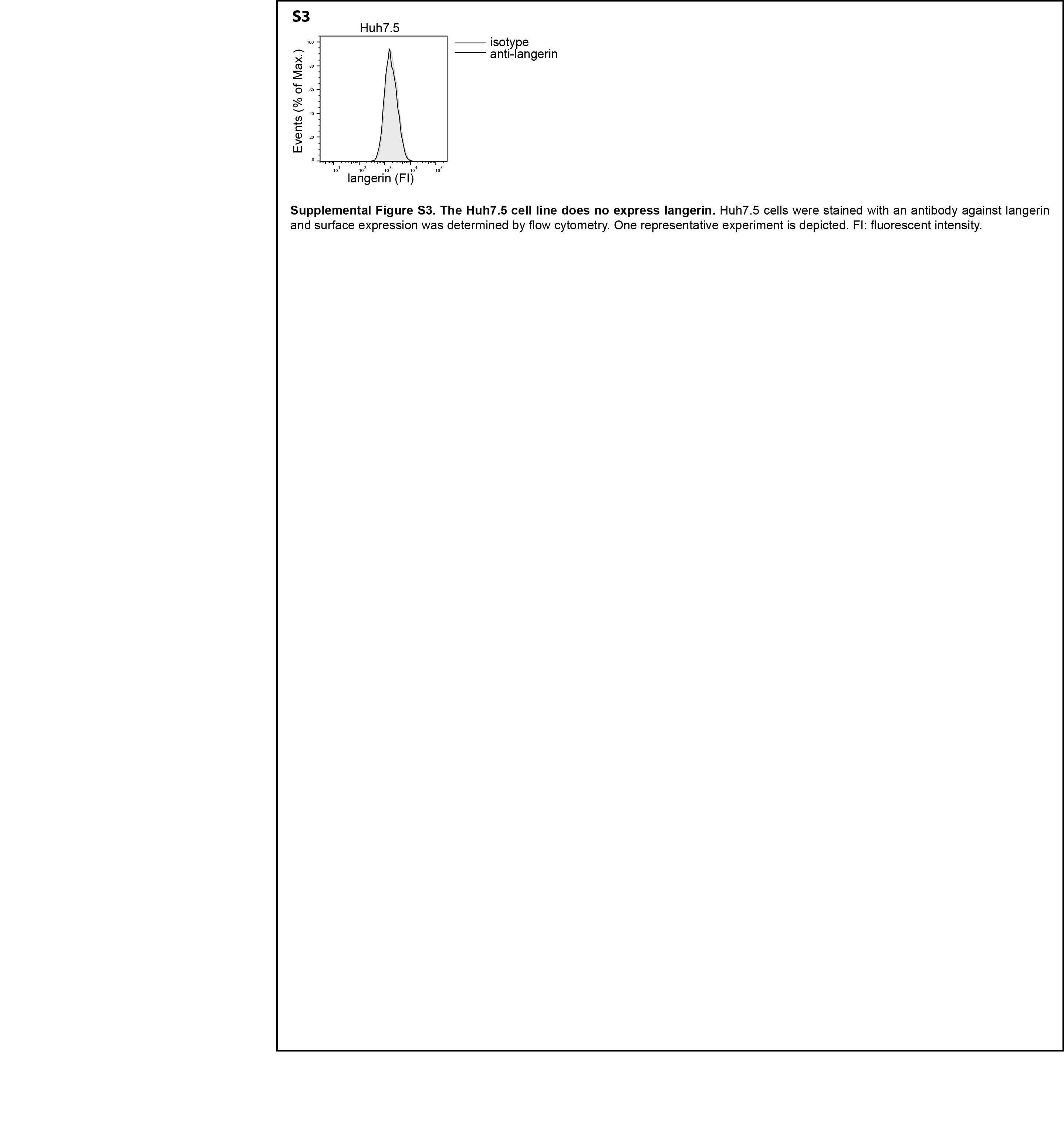

Supplement: Supplementary file 3 [file Image_3.jpg]

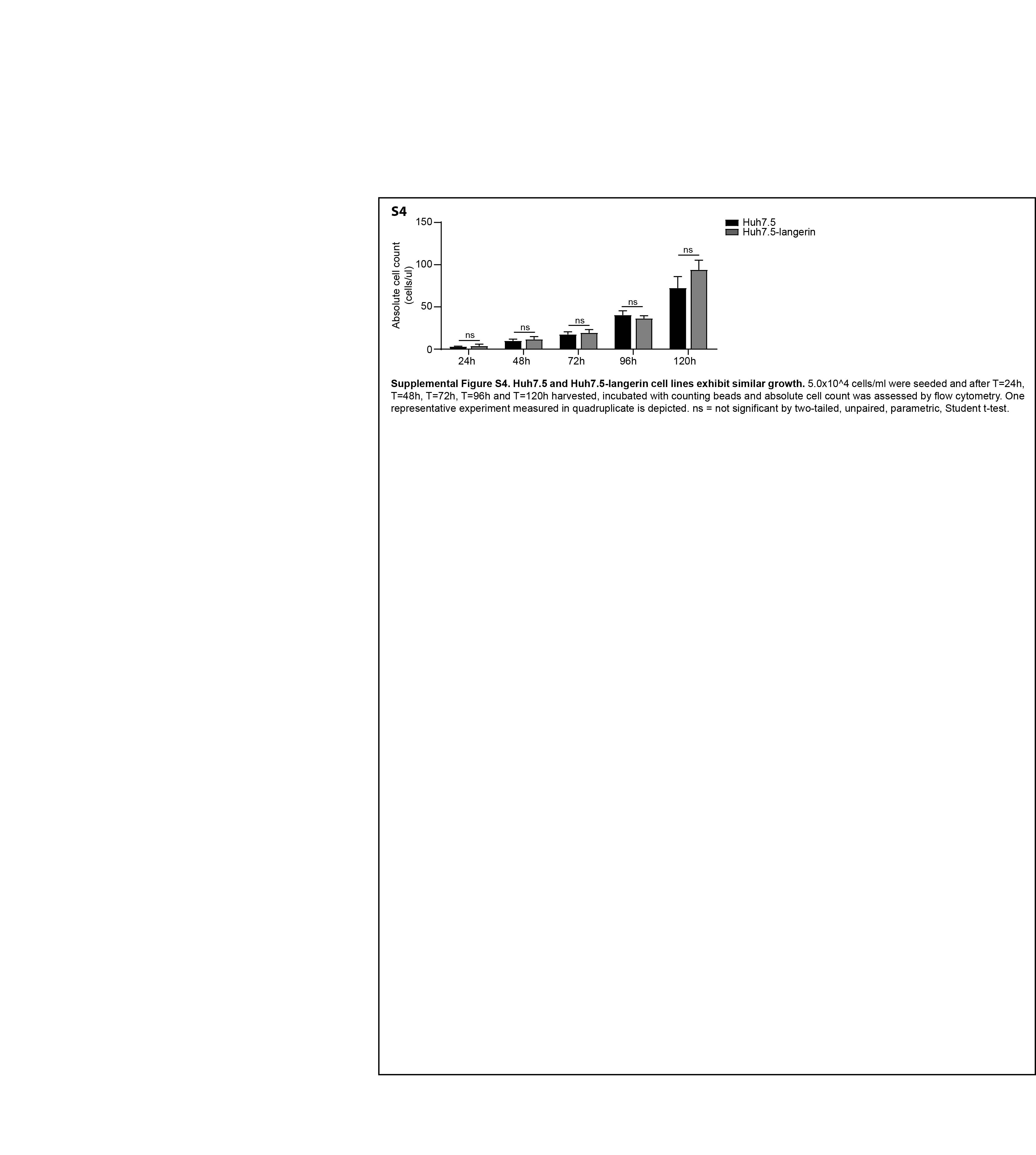

Supplement: Supplementary file 4 [file Image_4.jpg]

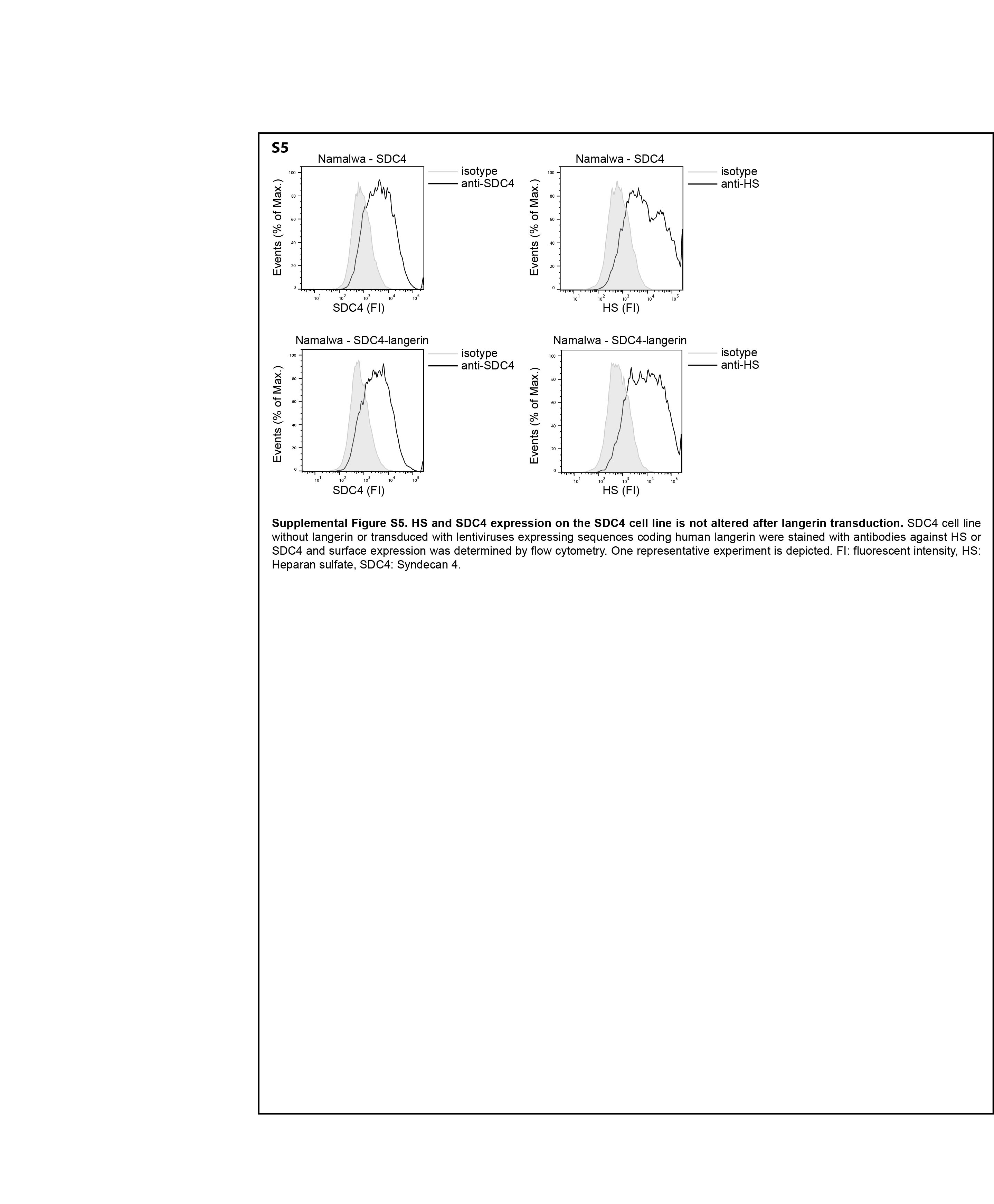

Supplement: Supplementary file 5 [file Image_5.jpg]
